# Developing AAV-delivered nonsense suppressor tRNAs for neurological disorders

**DOI:** 10.1016/j.neurot.2024.e00391

**Published:** 2024-07-02

**Authors:** Jiaming Wang, Guangping Gao, Dan Wang

**Affiliations:** a Horae Gene Therapy Center, University of Massachusetts Chan Medical School, Worcester, MA 01605, USA; b Department of Microbiology and Physiological Systems, University of Massachusetts Chan Medical School, Worcester, MA 01605, USA; c RNA Therapeutics Institute, University of Massachusetts Chan Medical School, Worcester, MA 01605, USA

**Keywords:** AAV, Nonsense mutation, Neurological disorder, Premature termination codon, Suppressor tRNA

## Abstract

Adeno-associated virus (AAV)-based gene therapy is a clinical stage therapeutic modality for neurological disorders. A common genetic defect in myriad monogenic neurological disorders is nonsense mutations that account for about 11% of all human pathogenic mutations. Stop codon readthrough by suppressor transfer RNA (sup-tRNA) has long been sought as a potential gene therapy approach to target nonsense mutations, but hindered by inefficient *in vivo* delivery. The rapid advances in AAV delivery technology have not only powered gene therapy development but also enabled *in vivo* preclinical assessment of a range of nucleic acid therapeutics, such as sup-tRNA. Compared with conventional AAV gene therapy that delivers a transgene to produce therapeutic proteins, AAV-delivered sup-tRNA has several advantages, such as small gene sizes and operating within the endogenous gene expression regulation, which are important considerations for treating some neurological disorders. This review will first examine sup-tRNA designs and delivery by AAV vectors. We will then analyze how AAV-delivered sup-tRNA can potentially address some neurological disorders that are challenging to conventional gene therapy, followed by discussing available mouse models of neurological diseases for *in vivo* preclinical testing. Potential challenges for AAV-delivered sup-tRNA to achieve therapeutic efficacy and safety will also be discussed.

## Introduction

Neurological disorders are diverse conditions afflicting the central and peripheral nervous systems. Although the etiology of neurological disorders can be environmental (e.g., exposure to toxins, malnutrition, or mechanical injury to nervous systems), DNA variants are the most common cause. For example, Rett syndrome is caused by *MECP2* mutations and spinal muscular atrophy is caused by *SMN1* mutations. In some cases, such as Alzheimer's disease (AD) and Parkinson's disease (PD), both environmental and genetic factors contribute to the disease state. To treat neurological disorders caused by known gene mutations, gene therapy is a straightforward and potentially effective strategy [[1], [2], [3], [4], [5], [6]]. Gene therapy was formally proposed in 1972 to address the genetic root cause of a disease by supplying a functional copy of the mutated gene [7]. Currently, recombinant adeno-associated virus (rAAV) is the leading *in vivo* gene therapy delivery platform [8,9], especially for neurological disorders that collectively have a high unmet medical need [10,11]. The first FDA-approved AAV gene therapy product for neurological disorders is Zolgensma, a single-dose AAV9-delivered *SMN1* for treating type 1 spinal muscular atrophy [12,13]. Many other AAV-based gene therapies for neurological disorders are under pre-clinical or clinical development [8,10,14].

Although traditional rAAV-based gene replacement therapy (i.e., replacing the mutated endogenous gene with a functional copy) holds tremendous promise for treating various neurological disorders, several drawbacks remain to be addressed. One concern is that the transgene can be overexpressed above physiological level or in off-target cell types, which may cause toxicity [[15], [16], [17]]. An alternative strategy is gene editing commonly mediated by CRISPR-based technologies, which can directly repair the mutated gene and harness the endogenous gene expression regulation. However, the large size and immunogenicity of CRISPR-associated (Cas) proteins pose challenges toward *in vivo* delivery [18,19].

Nonsense mutations, which account for about 11% of gene alterations responsible for inherited human genetic diseases [20], are DNA mutations in the protein-coding region that change a sense codon to a premature termination codon (PTC) in mRNA. Most PTC-containing mRNA will be degraded by nonsense-mediated mRNA decay (NMD), a mRNA quality control mechanism [21]. Translation of the remaining PTC mRNA is non-productive, because the PTC prematurely terminates protein synthesis to produce a truncated protein that is unfunctional or exerts dominant-negative effects. 18 out of 61 sense codons can be mutated to a stop codon via a single-nucleotide substitution. Among 50,574 nonsense mutations documented in the Human Genome Mutation Database (HGMD) (HGMD Professional release 2023.4), TAG (41.8%) is the most prevalent, followed by TGA (36.5%) and TAA (21.6%). When considering the wildtype (WT) amino acid residue, arginine codons to TGA (20.6%) and glutamine codons to TAG (18.8%) are the most common nonsense mutations (Table 1).

**Table 1:**
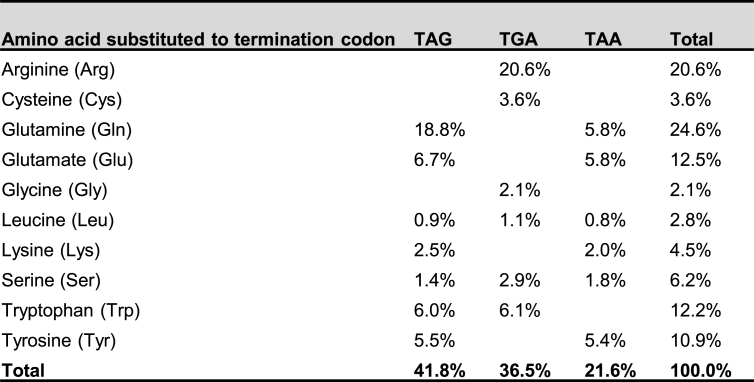
Survey of human pathogenic nonsense mutations. Human Genome Mutation Database (HGMD) (HGMD Professional release 2023.4).

CRISPR/Cas-mediated DNA editing technologies, such as base editing [22] and prime editing [23], can precisely correct a nonsense mutation. In addition, the PTC (UAA, UAG, or UGA) derived from a nonsense mutation can be corrected at the mRNA level in several ways. Adenosine deaminase acting on RNA (ADAR) catalyzes adenosine deamination to inosine, which is read as a guanosine during translation, thereby converting a PTC to a sense codon [24,25]. A box H/ACA ribonucleoprotein (RNP) converts the uridine in any PTC to pseudouridine (Ψ) [[26], [27], [28], [29]]; the resulting ΨAA codes for glutamine and tyrosine, ΨAG codes for glutamine, and ΨGA codes for arginine. These DNA/RNA editing technologies [30] are designed to specifically target a nonsense mutation or PTC by recognizing the flanking sequences, which greatly reduces the risk of off-target editing. However, developing therapeutics for individual nonsense mutations would require testing the efficacy and safety of numerous mutation-specific molecules.

The mutation-agnostic PTC readthrough therapy is an appealing treatment strategy to target multiple indications caused by a common PTC (UAA, UAG, or UGA) [31]. Use of small molecules as therapeutic readthrough agents was reviewed recently [[32], [33], [34], [35], [36]]. Notably, the aminoglycoside antibiotics gentamicin was tested in clinical trials, but nephrotoxicity and ototoxicity prevented its long-term administration [37,38]. Ataluren (also known as PTC124) was well tolerated and conditionally approved by the European Medicines Agency (EMA) in 2014 to treat nonsense-mediated Duchenne muscular dystrophy [39]. Unfortunately, post-authorization phase 3 studies failed to meet the primary endpoint, leading to EMA recommendation against renewal of Ataluren's marketing authorization in 2023 [40,41]. Ataluren remains an investigational drug in US due to FDA's concern over efficacy [42]. Therefore, there is a clear unmet medical need to develop better PTC readthrough therapies. Overall, small molecule readthrough compounds have relatively low readthrough activity, and their interaction with ribosome may impact multiple steps of translation besides termination.

Another class of PTC readthrough agent is suppressor transfer RNA (sup-tRNA). Sup-tRNA is derived from a natural tRNA with the anticodon altered to base-pair with one of three PTCs, and transfers the cognate amino acid to the elongating peptide, thereby restoring the production and function of the full-length protein [[43], [44], [45], [46], [47]]. Sup-tRNA therapeutics is well suited for AAV delivery due to its small gene size [46]. In this review, we discuss the potential therapeutic applications of AAV-delivered sup-tRNAs for neurological disorders, and challenges toward clinical translation.

## Sup-tRNA as Therapeutics Targeting Nonsense Mutations

Transfer tRNA (tRNA) is an adaptor molecule that decodes the genetic information in mRNA to the amino acid sequence of proteins. As the physical link between nucleic acid and protein, tRNA is responsible for maintaining the fidelity of genetic information. Meanwhile, it holds the potential to be engineered to recode the “wrong” genetic information caused by gene mutations.

### Discovery of sup-tRNA

In the early genetic studies on phage T4 and *E. coli*, certain phage T4 strains with rII nonsense mutations could not grow in the WT *E. coli* strain KB, but were rescued in another *E. coli* strain KB-3, indicating the presence of a suppressor activity to the nonsense mutations in the latter strain [48]. Similar suppression activity was observed in the *E. coli* strain *F-* with a suppressor activity, which could restore the phosphatase enzyme activity of another *E. coli* strain *Hfr* with the phosphatase nonsense mutation *P-* [49]. One hypothesis was that the suppressor activity came from a new or modified tRNA (also called “sRNA” that was just found to function as an adaptor between mRNA and peptide synthesis at that time [50]). Later, two studies independently proved this hypothesis: tRNA extracted from *E. coli* strains with suppressor activity could restore the synthesis of full-length protein in vitro in a cell-free system [51,52]. Suppressor tRNAs were also found in yeast [53], *C. elegans* [54], *Drosophila* [55], plants [56], and mice [57]; they could restore full-length protein synthesis or rescue the phenotype caused by nonsense mutations.

### Therapeutic applications of sup-tRNA

The discovery of the naturally occurring sup-tRNAs led to the idea that they can be harnessed as gene therapeutics for diseases caused by nonsense mutations. In an early attempt to demonstrate efficacy of sup-tRNA, a human natural tRNA^Lys^ was engineered to UAG-sup-tRNA^Lys^ via anticodon change, and co-delivered with β^0^ thalassemia mRNA containing a UAG PTC into Xenopus oocyte. 20 ​h later, the full-length β globin protein was detected [43]. Many ensuing studies have been focusing on the mechanisms of sup-tRNA-mediated readthrough, only a few aiming to adapt sup-tRNAs for therapeutic use [58,59]. These therapy-oriented studies informed on the potential of sup-tRNA therapeutics, and some exemplary ones are summarized and discussed below.

Nearly half of hereditary diffuse gastric cancer (HDGC; OMIM #137215) cases are caused by inherited germline mutations in *CDH1*, which encodes the E-cadherin protein [60,61]. *CDH1* germline mutation carriers have a high risk of gastric cancer due to somatic inactivation of the other WT allele. Among these mutations, nonsense mutations account for about 20% [62]. To develop sup-tRNA therapy for *CDH1* nonsense mutations, the anticodon of a tRNA^Arg^ (UCG) was mutated to UCA to decode UGA [63]. Co-delivery of plasmids expressing the UGA-sup-tRNA^Arg^ and nonsense *CDH1-R335X* cDNA (CGA→TGA), respectively, restored full-length CDH1 protein synthesis in three cell lines, AGS, MDA-MB-231, and CHO cells. The resulting CDH1 protein had normal membrane localization and could recruit β-catenin and p120-catenin to form adherens junction complex [63]. A plasmid containing five copies of sup-tRNA^Arg^ gene was created to enhance expression, but showed comparable readthrough efficiency compared with the single-copy design, suggesting that the sup-tRNA^Arg^ was potent and achieved saturated reporter readthrough level under the experimental condition.

Duchenne muscular dystrophy (DMD; OMIM #137215) is an X-linked recessive myopathy caused by mutations in the large *DMD* gene that encodes dystrophin [64]. Nonsense mutations account for 10–15% of all identified mutations [65]. The *mdx* mouse carries a nonsense mutation in exon 23, Q995X (CAA→TAA), that abrogates full-length dystrophin expression, and is the most widely used DMD animal model [66,67]. A plasmid carrying a UAA-targeting sup-tRNA (UAA-sup-tRNA) gene was constructed and injected to *mdx* mouse muscle, leading to 2.5% of dystrophin-positive fibers; muscle function recovery was unfortunately not reported [68]. In another study, intramuscular injection of an AAV8 vector carrying two copies of UAA-sup-tRNA^Ser^ gene led to dystrophin detection two to eight weeks post-injection [69].

Another example is cystic fibrosis (CF, OMIM # 219700), a common autosomal recessive genetic disease caused by mutations to the cystic fibrosis transmembrane conductance regulator (*CFTR*) gene [70,71], among which 10% are nonsense mutations. Two TGA nonsense mutations, G542X and W1282X are the most common, totaling 3.8% of *CFTR* pathogenic mutations [72]. Co-delivering a UGA-sup-tRNA along with a nonsense *CFTR-G542X or -W1282X* cDNA into HEK293 ​cells efficiently restored the expression of full-length glycosylated CFTR [44]. The PTC suppression efficiency was comparable when the sup-tRNA was delivered by plasmid or as an in vitro transcribed (IVT) RNA molecule. Correspondingly, chloride conductance was recovered to more than 50% of WT level [44]. The readthrough capability of sup-tRNAs was further validated in gene-edited human bronchial epithelial (16HBEge) cell lines harboring common *CFTR* nonsense mutations. Delivery of a UGA-sup-tRNA^Leu^ rescued CFTR protein from the *CFTR-W1282X* allele up to 45% of WT level [45], which is well above the predicted therapeutic threshold for CF (15–30%) [73]. Unlike a nonsense cDNA reporter, the nonsense mutation in the genomic *CFTR* gene renders the PTC mRNAs susceptible to NMD, which limits readthrough-induced CFTR protein restoration. Importantly, PTC readthrough by sup-tRNA was able to antagonize NMD and stabilize the *CFTR-W1282X* mRNA [45]. Another study employed rational design to engineer sup-tRNA body sequence for high PTC readthrough efficiency [47]. Delivery of IVT UGA-sup-tRNA^Arg^ variant tRT5 into human nasal epithelial (hNE) cells derived from CF patients homozygous for the R1162X mutation could effectively augment ion transport up to 14% of wildtype activity [47]. These encouraging studies using cell cultures warrant further investigation into the *in vivo* efficacy and safety of sup-tRNA for CF caused by nonsense mutations.

Hurler syndrome (OMIM # 219700) is one of the mucopolysaccharidoses caused by homozygous or compound heterozygous mutations in the *IDUA* gene encoding alpha-L-iduronidase, a lysosomal enzyme participating in the degradation of dermatan sulfate and heparan sulfate [74,75]. The *IDUA-W402X* (TGG→TAG) nonsense mutation is the most common mutation found in Hurler syndrome patients. In a mouse model carrying the *Idua-W401X* (TGG→TAG) mutation [76], rAAV-delivered sup-tRNA^Tyr^ safely and efficiently rescued the disease phenotype via synergistic PTC readthrough and NMD inhibition; therapeutic efficacy lasted for six months, a predetermined study endpoint, following a single systemic dosing [46]. The sup-tRNA could function in various tissues including the liver, heart, muscle, and brain, through optimization of AAV capsid and the route of administration for efficient gene delivery. Importantly, no gross toxicity was observed by histological and clinical serum biochemistry analysis. At the molecular level, ribosome profiling and tRNA sequencing demonstrated that rAAV.sup-tRNA^Tyr^ had a mild effect on transcriptome-wide readthrough at normal termination codons (NTCs) and endogenous tRNA homeostasis [46].

### Sup-tRNA engineering to enhance potency

**Fig. 1:**
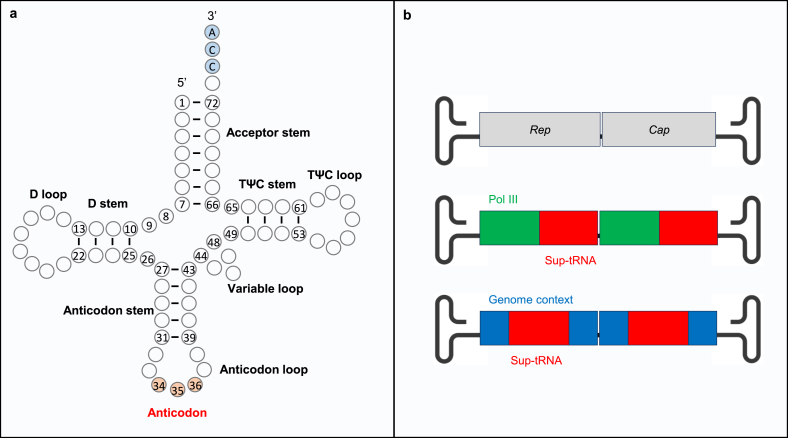
**tRNA secondary structure and AAV vector genome design to express sup-tRNAs.** (a) Secondary structure of typical mammalian cytosolic tRNAs. The CCA at the 3′ end is post-transcriptionally added and highlighted in blue; the anticodon is highlighted in orange. (b) The viral genome of WT AAV (top), and rAAV vector genomes containing two copies of a sup-tRNA gene driven by a Pol III promoter (middle) or in its natural sequence context (bottom). DNA length is not drawn to scale.

Readthrough ability is a critical attribute of sup-tRNA as therapeutics. A more potent sup-tRNA would achieve a higher level of full-length protein restoration and hold the potential to target more diseases requiring higher therapeutic thresholds. It may also enable a reduced dose and lower the potential toxicity associated with a high dose of delivery vehicle such as rAAV [77]. Most reported sup-tRNAs designed for PTC readthrough in mammalian cells only harbor sequence changes in the anticodon triplet to base pair with a stop codon, with a few exceptions that tinkered specific tRNA nucleotides outside anticodon [47,78]. Nevertheless, these studies suggest that the body sequence of tRNA is amenable to engineering (Fig. 1a), and can tolerate variations that synergize with an altered anticodon to enhance readthrough efficiency.

Rational design has been applied to engineering sup-tRNAs. In one study, a UGA-sup-tRNA^Arg^ variant carrying nucleotide substitutions in the TΨC stem was identified. This engineered new variant (i.e., tRT5) exhibited 1.5-fold higher readthrough ability than the parental sup-tRNA in a human CF bronchial epithelial cell line expressing full-length *CFTR* cDNA harboring nonsense mutations [47]. Another variant named tSA1T5 was derived from a UGA-sup-tRNA^Ser^. Co-delivering tSA1T5 and a PTC-containing mRNA reporter (both were generated via IVT) to mice showed that tSA1T5 induced high readthrough in the liver and lung [47]. Besides the body sequence, the sequences flanking mature sup-tRNA, including the 5′ upstream control element and 3’ trailer sequences were recently interrogated for their impact on readthrough activity using a luciferase reporter assay [79]. The optimized sequence context increased sup-tRNA expression, leading to higher nonsense suppression activity. However, deriving a generalizable rule to optimize the flanking sequences is difficult due to their potential interplay with individual sup-tRNAs [79].

It is worth noting that a range of attributes of sup-tRNAs and engineered variants should be carefully analyzed, because sequence changes in natural tRNAs may impact their interaction with cognate aminoacyl-tRNA synthetase (aaRS) and amino acid charging. For example, UAG-sup-tRNAs derived from human tRNA^Trp^ and tRNA^Gln^ via anticodon modification were mischarged by lysine at a frequency of 79% and 23%, respectively [46]. In another study, a UGA-sup-tRNA^Trp^ harboring changes in both anticodon and the TΨC-stem was charged with arginine instead of tryptophan [79]. Besides charging fidelity, the stability and integrity of mature sup-tRNA can also impact therapeutic efficacy. It is increasingly recognized that tRNA-derived fragments (tRFs) are naturally generated from endogenous tRNAs and involved in many cellular processes [[80], [81], [82]]. Sup-tRNAs may generate unique tRF species that exert undesired biological effects [[83], [84], [85]]. With the advances in sequencing technologies to measure tRNA and tRFs [[86], [87], [88], [89], [90], [91], [92], [93]], more comprehensive characterization of therapeutic sup-tRNAs is becoming attainable.

## Recombinant Adeno-Associated Virus (rAAV) Delivery of Sup-tRNA

As for other nucleic acid-based therapeutics, delivery has been a major challenge for sup-tRNA to achieve *in vivo* therapeutic efficacy and durability. Currently, lipid nanoparticle (LNP) and rAAV are the major *in vivo* nucleic acid delivery platforms, and both have been attempted for sup-tRNA applications [46,47]. Sup-tRNA can be generated by IVT and packaged in LNPs for *in vivo* delivery. IVT sup-tRNAs lack base modifications that exist in natural tRNAs and play important roles in tRNA stability, function, and immunogenicity. Nevertheless, several studies showed that LNP-sup-tRNAs induced efficient PTC readthrough in cells and in mice, showing that IVT sup-tRNAs can be charged once enter cells and participate in translation [47]. Although LNP-sup-tRNA will only afford transient PTC readthrough due to sup-tRNA turnover, it may allow repeated dosing. Alternatively, sup-tRNA genes can be delivered by rAAV, so that proper base modifications can be deposited to sup-tRNA during its cellular biogenesis. The rAAV vector genome remains as stable episomal DNA in cells allowing for long-term expression, which is a desired feature for RNA-targeted therapy such as sup-tRNA. Several rAAV-based gene therapies for neurological disorders are in the clinical stage [8,10,14]. Here, we focus on rAAV delivery of sup-tRNA. Interested readers are encouraged to refer to other literature on LNP delivery [47,59,94,95].

### rAAV for sup-tRNA delivery

AAV is a small single-stranded (ss) DNA virus belonging to the *Dependoparvovirus* genus within the *Parvoviridae* family [96]. The ss viral genome is ∼4.7 ​kb and flanked by two T-shaped inverted terminal repeats (ITR), serving as the viral origins of replication and packaging signal [97]. The *Rep* gene encodes four replicase proteins required for viral replication [98], and the *Cap* gene encodes three structural proteins (VP1, VP2, VP3) that form the viral capsid of ∼26 ​nm in diameter [99,100]. Another two proteins, assembly activating protein (AAP) and membrane-associated accessory protein (MAAP), are encoded within the *Cap* coding sequence but in different reading frames, and involved in virion assembly and egress, respectively [[101], [102], [103], [104]]. In rAAV genome, the majority of WT viral DNA is replaced by a desired gene expression cassette to produce therapeutic RNA and/or protein following cellular delivery, leaving only the two ITRs supporting genome replication and rAAV packaging [105,106]. The ITR-flanked rAAV genome can be manipulated using conventional molecular cloning methods. Importantly, the total rAAV genome size shall not exceed 5.0 ​kb for proper packaging. Once delivered to the nucleus, the ss rAAV genome needs to be converted to a double-stranded (ds) form to allow for transcription [107,108]. To bypass this rate-limiting step for transgene expression, the self-complementary (sc) rAAV genome was designed using a Rep-nicking resistant mutant ITR [109]. Compared with the ss rAAV genome, the sc configuration enables faster and higher transgene expression [109,110]. Although the packaging capacity of a sc rAAV genome is halved to 2.5 ​kb, it is not a major concern for sup-tRNA gene delivery due to its small gene size.

rAAV9 was used to deliver two copies (2×) of UAG-sup-tRNA^Tyr^ gene to a nonsense *Idua* knock-in (KI) mouse model of Hurler syndrome [46]. Although intravenous (IV) delivery of rAAV9.2×sup-tRNA^Tyr^ significantly restored IDUA activity in the liver and heart, the skeletal muscle and brain were less responsive, showing only 0.5% of WT IDUA activity in muscle and no detectable restoration in the brain. IV delivery by a more muscle-tropic capsid AAVMYO [111] under the same condition as AAV9 treatment restored IDUA activity in the muscle to 7% of the WT level. The same construct was also intravenously delivered by AAV.PHPeB, which is an engineered capsid that crosses the blood–brain barrier (BBB) in certain mouse strains 100-fold more efficiently than AAV9 [112]; IDUA activity in the brain was restored to 1.3% of the WT level. In addition to using more potent capsid, optimizing the route of administration can also improve delivery and sup-tRNA efficacy. rAAV9.2×sup-tRNA^Tyr^ delivered by intramuscular injection restored IDUA activity in the injected muscle up to 5% of the WT level, and unilateral intrahippocampal injection resulted in an even higher IDUA activity in the injected hippocampus, reaching 10% of the WT level. This study demonstrated that the readthrough activity of sup-tRNA *in vivo* is highly dependent on the delivery efficiency [46].

One of the most striking advantages of rAAV-delivered gene therapy is its long-term efficacy [113,114]. In a long-term follow-up study of AVXS-101/Zolgensma phase 1 clinical trial, a favorable safety profile and durable efficacy were observed for up to 6.2 years after dosing [115]. In other studies, the efficacy of rAAV therapeutic vectors in animal models and human patients were shown to last more than 10 years [116,117]. However, transgene expression declines were also observed, presumably due to immune responses or vector dilution [[118], [119], [120]]. In some cases, repeated dosing may be required. Recent studies suggested that immunomodulation [121,122] and antibody-cleaving enzymes [[123], [124], [125]] could be viable strategies to enable rAAV re-dosing.

### Advances in developing neurotropic AAV capsids

The AAV capsid largely determines tissue tropism and delivery efficiency. The BBB poses a unique challenge to developing AAV capsids that can target the central nervous system (CNS) following systemic delivery [[126], [127], [128]]. In this regard, AAV9 is arguably the most efficient naturally occurring serotype [[129], [130], [131]], and generally considered as the benchmark when characterizing novel capsids. Building upon a panel of known AAV capsid serotypes, various capsid engineering approaches have been used to improve tropism.

Shuffling natural AAV capsids of different serotypes has yielded variants with new CNS tropism. For example, AAV.Olig001 was generated by shuffling AAV 1, 2, 5, 6, 8, and 9, and transduced >95% of striatal oligodendrocytes after intracranial infusion in rats [132]. AAV-B1 was derived from shuffling AAV 1, 2, 4, 5, 6, 8, 9, rh8, rh10, rh39, and rh43, and showed widespread gene transfer throughout the CNS following systemic injection in adult mice and cats [133]. Randomized peptide library insertion combined with a screening process has proven to be an effective AAV capsid engineering approach. Several potent BBB-crossing capsids were generated using this approach, such as AAV-PHP.B [134], AAV-PHP.eB [112], and AAV-F [135]. Notably, many of the BBB-crossing capsids identified in mice converge on exploiting LY6A as the receptor that is not conserved in primates [136,137]; therefore, the importance of capsid engineering in the context of non-human primate (NHP) for clinical translatability is increasingly recognized. Some species-agnostic AAV capsid engineering and/or screening methods have generated promising candidates to target NHP CNS much more efficiently than AAV9 [[138], [139], [140], [141], [142]].

### Route of administration of AAV vector

The choice of route of administration is an important consideration when targeting the CNS [10,14,[143], [144], [145]]. IV injection of BBB-crossing rAAV to target CNS is convenient, and can potentially achieve widespread delivery, which is advantageous for treating diseases afflicting the entire CNS. However, it often requires a high systemic dose compared with local injection, and may trigger deleterious immune responses [[146], [147], [148]]. Moreover, rAAV delivered via blood stream is susceptible to circulating pre-existing neutralizing antibodies present in general human populations [[149], [150], [151]]. rAAV delivered to the brain tissue or cerebrospinal fluid (CSF), such as by intracerebroventricular, intra-cisterna magna, or intrathecal delivery, leads to more CNS-restricted transduction compared with the systemic route. However, these direct injection methods are more invasive, and injection at multiple sites may be required to achieve sufficient spread. Although these CNS-restricted administration methods are less likely to trigger whole-body immune responses, local inflammation and astrocyte activation were observed after injection [152,153].

### Producing rAAV-sup-tRNA

**Fig. 2:**
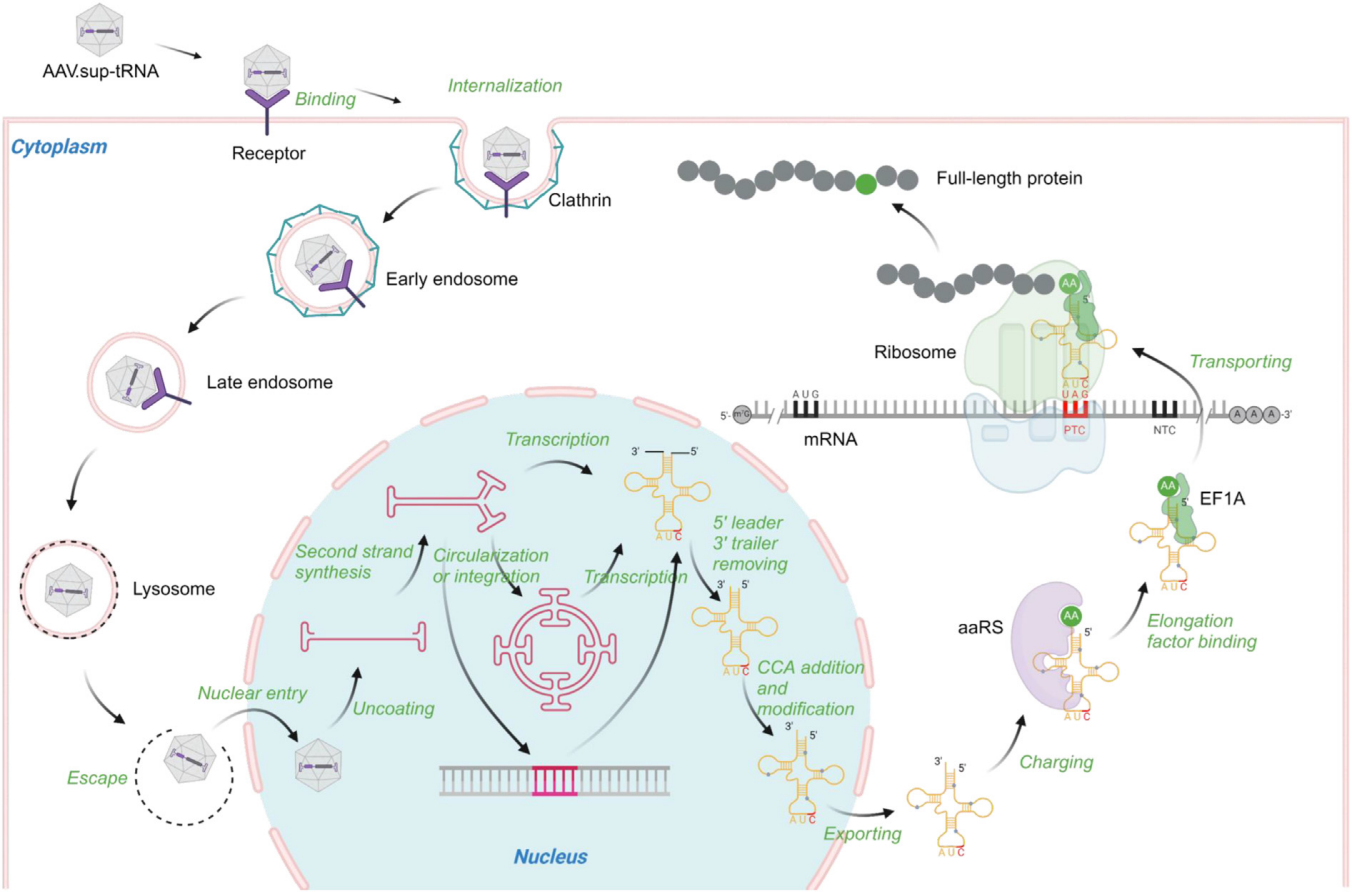
**Biogenesis and action of sup-tRNA delivered by rAAV.** rAAV is recognized by cell surface receptors, which triggers its internalization via clathrin-mediated endocytosis. Following endosomal escape, rAAV translocates into the nucleus and releases the single-stranded DNA genome following uncoating. Double-stranded and transcriptionally active vector DNA was formed mainly by second-strand synthesis, although a self-complementary vector genome can bypass this step. Inter-molecular or intra-molecular recombination between the viral inverted terminal repeats (ITRs) generates circularized episomal genomes that lead to stable transgene expression. The rAAV genomes may integrate into the host genome at a low frequency. The sup-tRNA gene is transcribed to generate pre-sup-tRNA, which undergoes several maturation steps including removing the leader sequence by RNase P and the trailer sequence by RNase Z, adding CCA by tRNA nucleotidyltransferase, removing introns in some tRNAs by tRNA splicing endonuclease (TSEN) complex, and post-transcriptional modifications by various enzymes. Once exported to the cytoplasm, sup-tRNA is charged with amino acid by aminoacyl-tRNA synthetase (aaRS). Aminoacyl-sup-tRNA is delivered to the ribosomal A-site by the elongation factor eEF-1A, where it decodes the premature termination codon (PTC) with the modified anticodon, and inserts an amino acid into the nascent peptide.

Several rAAV manufacturing platforms have been developed, including transient triple plasmids transfection in HEK293 ​cells, baculovirus-mediated infection in insect cells, and stable packaging or producer cell lines; each of these methods has advantages and drawbacks in terms of flexibility, vector yield, vector quality, and scalability [154]. The most common method to produce rAAV is co-transfection of three plasmids to HEK293 ​cells at roughly equal molar ratio: the cis plasmid carrying the ITR-flanked transgene (pCis), the trans plasmid that expresses Rep and Cap (pTrans), and the helper plasmid that expresses certain adenoviral genes (pHelper) [[155], [156], [157]]. It is straightforward to generate pCis carrying a sup-tRNA expression cassette using standard molecular cloning techniques. As in natural tRNA genes, a sup-tRNA gene (∼70 bp or ∼155 bp with flanking sequences [58]) harbors intrinsic Pol III promoter activity that drives its own expression. An external Pol III promoter, such as the U6 promoter (∼250 bp), can be placed upstream of the sup-tRNA gene to boost expression. In either design, the small transgene sizes fit well within the packaging limit of AAV (Fig. 1b). Once successfully packaged into rAAV, the sup-tRNA gene can be delivered to cells carrying nonsense mutation. Following transcription and maturation, the sup-tRNA is charged with amino acid and decodes the PTC with the modified anticodon (Fig. 2).

However, some difficulties in packaging sup-tRNA genes into rAAV were noted. In one study, standard triple transfection failed to package UGA-sup-tRNA genes, because UGA-sup-tRNA expressed in HEK293 ​cells compromised Rep and Cap expression. Surprisingly, reducing the amount of pCis to 1% allowed for Rep and Cap expression and afforded successful rAAV-sup-tRNA production [158]. In another study, multimeric sup-tRNA cassettes were employed to enhance sup-tRNA expression and readthrough efficiency. Unexpectedly, rAAV9 harboring four copies of UAG-sup-tRNA^Tyr^ (rAAV9.4×sup-tRNA^Tyr^) showed lower efficacy than rAAV9.2×sup-tRNA^Tyr^. Further investigation revealed a considerable amount of truncated vector genomes in rAAV9.4×sup-tRNA^Tyr^, likely caused by the extensive repeating sequences [46].

## Neurological Disorders Amenable to Sup-tRNA Therapy

Sup-tRNA is particularly suitable for treating genetic diseases that exhibit several features including: (1) there exists a high frequency of nonsense mutations in the disease-causing gene; (2) overexpression of the therapeutic gene would be toxic; (3) the cDNA gene size is too large to fit AAV packaging limit (∼5.0 ​kb). Exemplary neurological diseases that meet one or more of these criteria are shown in Table 2 and discussed as follows.

**Table 2:**
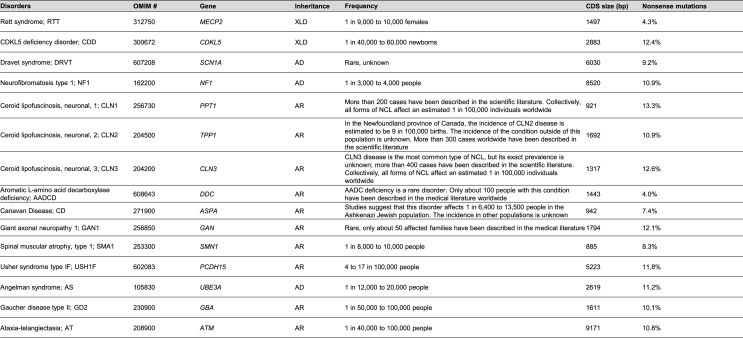
A selected list of neurological disorders amenable to sup-tRNA therapy. MECP2, methyl-CpG-binding protein-2; CDKL5, Cyclin-dependent kinase-like 5; SCN1A, Sodium voltage-gated channel alpha subunit 1; NF1, Neurofibromin1; PPT1, Palmitoyl-protein thioesterase 1; TPP1, Tripeptidyl peptidase 1; CLN3, CLN3 lysosomal/endosomal transmembrane protein, battenin; DDC, Dopa decarboxylase; ASPA, Aspartoacylase; GAN, Gigaxonin; SMN1, Survival of motor neuron 1; PCDH15, Protocadherin related 15; UBE3A, Ubiquitin protein ligase E3A; GBA, Glucosylceramidase beta 1; ATM, ATM serine/threonine kinase. The percentage of nonsense mutations within all pathogenic mutations are from Human Genome Mutation Database (HGMD) (HGMD Professional release 2023.4). Frequency of disorders are from MedlinePlus (https://medlineplus.gov/) (01/25/2024). XLD: X-linked dominant. AD: autosomal dominant. AR: autosomal recessive. CDS: coding sequence.

**Fig. 3:**
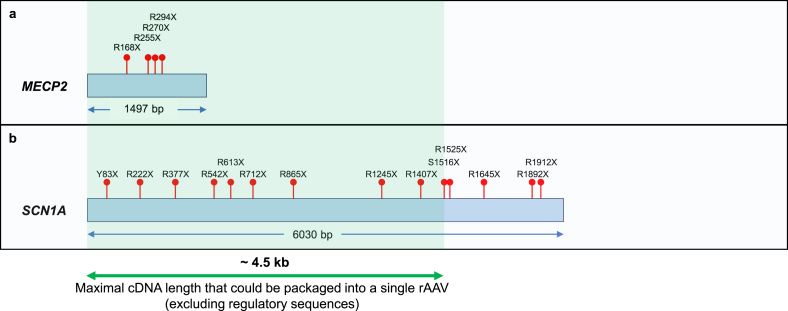
**Pathogenic nonsense mutations in representative genes causing neurological disorders.** Complementary DNAs (cDNAs) of *MECP2* and *SCN1A*, and their recurrent nonsense mutations identified in patients are shown to scale. The *SCN1A* cDNA exceeds the maximal DNA size (∼4.5 ​kb, excluding regulatory sequences) that can be packaged into rAAV (double-headed green arrow).

Rett syndrome (RTT, OMIM #312750) is an X-linked neurodevelopmental disorder that affects 1 in 10,000 female births. The typical RTT patients show arrested neurological development at the early stage of life accompanied by symptoms including loss of speech, impaired motor skills, stereotypical hand movements, gait abnormalities, and seizures. About 95% of patients with typical RTT carry mutations in the *MECP2* gene encoding methyl-CpG-binding protein 2, a transcription regulator [[159], [160], [161], [162]]. Extensive animal studies suggest that gene therapy may be a viable treatment strategy. First, RTT was attributable to the loss of MECP2 in the nervous system [[163], [164], [165], [166]], pointing to the relevant target tissue for gene delivery. Second, MECP2 function is required in adult animals [167,168], suggesting that restoring MECP2 later in life can be beneficial. Third, congenital MECP2 deficiency can be rescued in adult animals by genetic means [169], demonstrating reversibility of the disease course. However, AAV gene replacement therapy development for RTT has proven challenging, largely because unregulated overexpression of MECP2 is harmful [170]. The deleterious consequence of supraphysiological MECP2 expression is also seen in patients of *MECP2* duplication syndrome (MDS) [171,172]. In contrast, sup-tRNA operates on PTC-containing endogenous *MECP2* mRNA that is subjected to native transcriptional regulation, thereby restoring protein level within the normal range. Furthermore, nonsense mutations account for ∼30% of RTT cases, and most are arginine codons to TGA [160,161,173] (Fig. 3a). Therefore, a single sup-tRNA that installs arginine at a UGA PTC (UGA-sup-tRNA^Arg^) can restore WT MECP2 and treat a large proportion of patients.

Dravet syndrome (DRVT, OMIM #607208) is a severe early-onset developmental and epileptic encephalopathy associated with cognitive, motor, and behavioral impairments [174,175]. Anti-seizure medications are the most common treatment that can reduce seizures but leaving multiple co-morbidities unaddressed. 80% of Dravet syndrome patients harbor mutations in *SCN1A* that encodes sodium channel subunit NaV1.1 [[176], [177], [178], [179]]. Several transcriptional activation therapies aiming to increase functional NaV1.1 expression are currently under development [180]. AAV-mediated gene replacement therapy is hindered by the large *SCN1A* cDNA size (6030 bp) (Fig. 3b). Considering that nonsense mutations represent ∼20% of *SCN1A* gene lesions [178], sup-tRNA can benefit a sizable patient population.

Many monogenic neurological disorders are potentially treatable by traditional AAV gene replacement therapy or gene editing therapy. However, the small patient population of individual rare and ultrarare genetic diseases poses unique challenges to gene therapy development, such as difficulty in conducting clinical trials and lack of commercial viability. Sup-tRNA can potentially target multiple unaddressed indications with a common PTC, and enable a one-size-fits-many gene therapy paradigm. Future clinical studies may recruit patients sharing the same PTC across multiple diseases, and adopt the basket trial design in oncology to streamline translation [181,182]. Therefore, sup-tRNA may be a viable gene therapy solution for a large population of patients suffering from diverse CNS pathologies but sharing a common PTC [183,184].

## Cellular and Animal Disease Models for Therapeutic Study

Testing the therapeutic effect of sup-tRNAs requires proper disease models carrying nonsense mutations. *In vitro* cell models are useful for the initial high-throughput screening and validation of sup-tRNAs, whereas disease animal models can greatly facilitate the preclinical efficacy and safety assessment. Mice are the most used mammalian model organism in biomedical research. In this section, we discuss commonly used cellular models and available mouse models to test sup-tRNA therapeutic efficacy.

### Cell models for sup-tRNA study in vitro

**Fig. 4:**
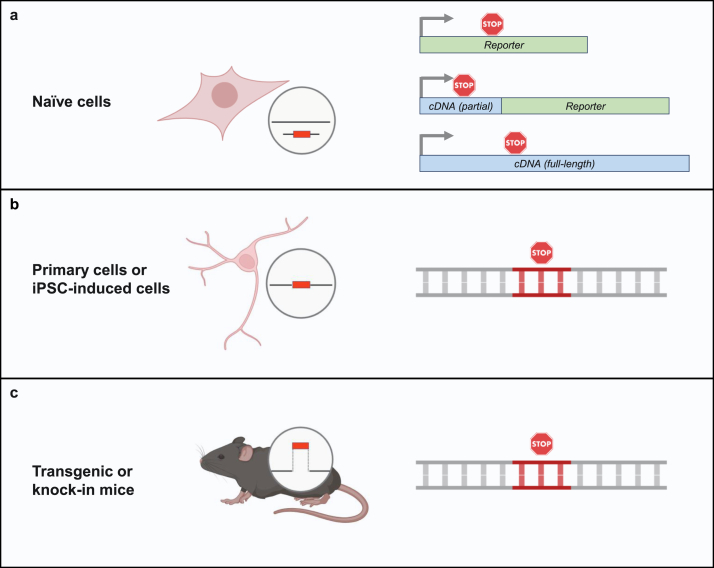
**Cellular and animal disease models for sup-tRNA readthrough study.** (a) Naïve cells transfected with a mutant reporter gene disrupted by a nonsense mutation (stop sign). The nonsense mutation may be in a reporter gene, or in the sequence context of a disease-associated partial or full-length cDNA. (b) Primary cells or iPSC-derived cells carrying a nonsense mutation in an endogenous disease-causing gene. (c) Genetically modified mouse harboring a native, genomic nonsense mutation in an endogenous gene.

Two types of cellular assays are commonly used to test sup-tRNA function: 1) co-delivering a sup-tRNA and an exogenous nonsense reporter to cells (Fig. 4a), and 2) delivering sup-tRNA to cells that carry a genomic pathogenic nonsense mutation (Fig. 4b).

In the first approach, sup-tRNA-mediated PTC readthrough rescues the nonsense reporter, leading to the expression of a protein that can be conveniently quantified, such as a fluorescent protein or luciferase; readthrough efficiency is calculated as the percentage of sup-tRNA-induced protein expression normalized to that of a WT reporter (i.e., 100% readthrough). Because the baseline PTC readthrough in the absence of sup-tRNA is usually highly variable and barely detectable, readthrough efficiency calculated as fold-change over baseline readthrough should be interpreted with caution. Both sup-tRNA and reporter can be delivered as plasmids or IVT RNAs. A body of literature demonstrated that the mRNA sequence context surrounding a stop codon can impact readthrough [[185], [186], [187], [188], [189]]. Therefore, the targeted disease-causing PTC placed in its natural cDNA sequence context, rather than a fluorescent protein or luciferase gene, serves as a more accurate reporter that better informs on readthrough at the disease PTC of interest. Even a carefully designed and controlled readthrough reporter assay suffers from intrinsic limitations. For example, sup-tRNA function in commonly used immortalized cell lines (e.g., HEK293) may not predict the *in vivo* readthrough efficiency, because tRNAs and aminoacyl-tRNA synthetases are subjected to various dysregulation under cancer conditions [[190], [191], [192], [193]]. In addition, while most endogenous pathogenic PTCs trigger NMD, the intron-less PTC-containing reporter mRNAs are immune to NMD and therefore may overestimate sup-tRNA potency.

Primary cells derived from patients or animal models harboring a native genomic nonsense mutation are a valuable platform to test sup-tRNA function. A large collection of patient cells is available from The Coriell Institute for Medical Research, such as fibroblasts and induced pluripotent stem cells (iPSCs). Furthermore, iPSCs can be differentiated into various CNS cell types, allowing for testing sup-tRNA function in specific cell types. These human-derived primary cells are generally refractory to nuclei acid transfection, but electroporation or viral vector infection may achieve sufficient sup-tRNA delivery [46]. The PTC readthrough efficiency can be gauged by full-length protein restoration using immunoassays (e.g., western blotting), and the resulting biological effects can inform on therapeutic efficacy.

### Disease mouse models for sup-tRNA study *in vivo*

While the majority of disease mouse models carry a knock-out allele, and therefore are not suitable to test sup-tRNA therapy, the advent of gene editing technology has greatly facilitated the generation of mouse models carrying specific mutations such as nonsense mutations (Fig. 4c). For the myriad neurological disorders caused by nonsense mutations in different genes, only a limited number of nonsense-mediated mouse models are available. However, testing sup-tRNAs for their PTC readthrough efficiency in these available mouse models may inform on their potential for treating other neurological diseases. It should be noted that mouse models have intrinsic limitations in recapitulating human disease and predicting clinical efficacy. In contrast, large animal models harboring nonsense mutations may serve as better alternatives but are scarcely available [[194], [195], [196], [197], [198], [199]] and require special veterinary expertise. Therefore, we focus our discussion on various mouse models that are widely accessible to researchers.

High-efficiency mutagens such as N-ethyl-N-nitrosourea (ENU) were widely used in the early forward genetic screens in mice. The DNA mutations induced by ENU are primarily point mutations that may result in nonsense mutations [200]. The *nur7* allele carrying a TAA nonsense mutation (c.C577T, p.Q193X) in the *Aspa* gene was identified in this way [201,202]. Homozygous *nur7* mice lack aspartoacylase that is normally highly expressed oligodendrocytes, and recapitulate a childhood leukodystrophy known as Canavan disease. This mouse model has been used in the preclinical gene therapy studies for Canavan disease [203], and may provide a useful tool to test UAA-sup-tRNA function in oligodendrocytes.

Targeted homologous recombination was the most effective means to generate mouse models prior to the wide adoption of gene editing [204]. This approach involves introducing a targeting vector harboring the desired genetic alteration along with a drug-resistance gene and a negative selectable marker into mouse embryonic stem cells (ESCs). Following homologous recombination between the targeting vector and the genomic locus, drug-resistant ESC clones are isolated, selected, and used to produce heterozygous mice. Several mouse lines carrying nonsense mutations were created to model neurological diseases that include Rett syndrome [[205], [206], [207]], Dravet syndrome [208,209], Neurofibromatosis type 1 (NF1) [210], CDKL5 deficiency disorder (CDD) (also known as developmental and epileptic encephalopathy-2) [211], Batten diseases (also known as neuronal ceroid lipofuscinoses) [212,213], and Hurler syndrome [76] (Table 3).

**Table 3:**
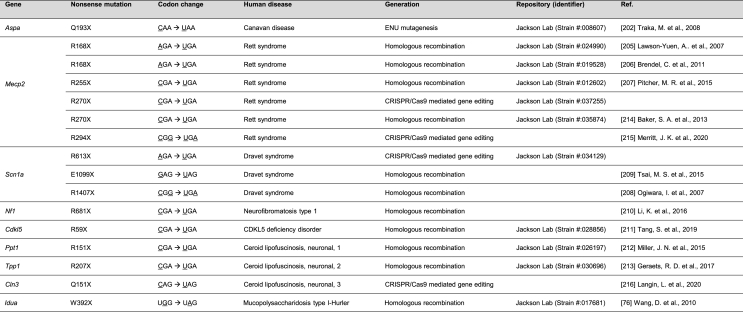
Representative disease mouse models harboring nonsense mutations.

Gene editing, especially CRISPR-based technologies, revolutionized animal modeling, and it is currently the mainstream approach to introducing small genetic changes such as a nonsense mutation. CRISPR-based gene editing can also accommodate larger changes, such as humanizing an entire exon (i.e., replacing an exon in a mouse gene with the human counterpart). Because the sequence context of a PTC has a profound impact on readthrough, placing the nonsense mutation in a humanized exon may better predict sup-tRNA function toward human mutation and streamline translation. CRISPR gene editing is increasingly used to generate nonsense mutation mouse models, some of which are relevant to neurological diseases and summarized in Table 3 [[214], [215], [216], [217]].

## Perspectives: Striking a Balance Between Efficacy and Safety

Developing sup-tRNA therapeutics is still at the early stage, awaiting more studies that demonstrate sufficient *in vivo* PTC readthrough and meaningful therapeutic efficacy in disease animal models. A body of literature on tRNA biology and sup-tRNA engineering suggest the feasibility to design more potent sup-tRNAs via optimizing the body sequences and flanking sequences [47,78,79,218]. To further enhance readthrough efficiency and/or protein restoration, sup-tRNA can be potentially combined with other strategies such as translation termination modulation and NMD inhibition, which proved to synergize with small-molecule readthrough drugs [[219], [220], [221], [222], [223], [224], [225], [226]].

As the on-target PTC readthrough increases, undesired NTC readthrough will likely ensue and pose a safety concern. The transcriptome-wide NTC readthrough of many endogenous mRNAs may generate C-terminus extended proteins that trigger immune responses and/or stress responses [220,227,228]. Some readthrough protein products may exert a gain-of-toxicity effect [229,230]. However, several ribosome profiling studies showed that NTC readthrough by sup-tRNAs was moderate or undetectable [44,46]. Furthermore, multiple endogenous mechanisms exist to mitigate deleterious NTC readthrough events. For example, multiple in-frame backup stop codons are enriched in the 3’ untranslated regions (UTR) of natural mRNAs [231,232], and mammalian cells possess quality control mechanisms to degrade aberrant proteins caused by NTC readthrough [227,228,[233], [234], [235]]. Nevertheless, it is possible that sup-tRNA-induced NTC readthrough overwhelms these safeguard mechanisms. Therefore, characterizing NTC readthrough and consequences upon sup-tRNA delivery is an important safety consideration toward therapeutic development.

The potential toxicity related to NTC readthrough may be specific to certain cell and tissue types, because their transcriptomes vary substantially. For neurological disorders, the toxicity originating from the target tissue (e.g., brain) may be dampened by lowering the dose of sup-tRNA to reach a balance between efficacy and tolerability. To mitigate sup-tRNA delivery to off-target tissues and the resulting toxicity, AAV capsid with tissue de-targeting property may be considered. For example, the liver toggle AAV9 variant (G-to-A amino acid change at residue 267 of VP1) was shown to greatly diminish rAAV9 delivery to the mouse and monkey liver, which may potentially improve the safety profile of CNS-targeted sup-tRNA delivery [236].

AAV-delivered sup-tRNA therapeutics can potentially address many currently incurable neurological disorders caused by nonsense mutations. The readthrough ability of sup-tRNA and rAAV delivery efficiency are important considerations in preclinical development, interrogation of which in *in vivo* disease animal models will inform on translatability. In addition, potential toxicity caused by NTC readthrough in the nervous system and other off-target tissue types should be carefully monitored to ensure an acceptable balance between safety and efficacy.

## Author Contributions

J.W. wrote the original draft. G.G. and D.W. revised the manuscript.

## Declaration of competing interest

The authors declare the following financial interests/personal relationships which may be considered as potential competing interests: Jiaming Wang, Guangping Gao, Dan Wang has patent pending to University of Massachusetts Chan Medical School. If there are other authors, they declare that they have no known competing financial interests or personal relationships that could have appeared to influence the work reported in this paper.
